# Acute effects of continuous and interval cycling on salivary SIgA and anti-microbial peptide secretions: a randomized crossover trial

**DOI:** 10.1007/s00421-025-05937-5

**Published:** 2025-08-14

**Authors:** Reita Ito, Masataka Uchida, Shumpei Fujie, Keiko Iemitsu, Chihiro Kojima, Yasushi Shinohara, Takeshi Hashimoto, Tadao Isaka, Motoyuki Iemitsu

**Affiliations:** 1 Faculty of Sport and Health Science, Ritsumeikan University, 1-1-1 Nojihigashi, Kusatsu, Shiga 525-8577 Japan; 2 Research Organization of Science and Technology, Ritsumeikan University, Kusatsu, Japan; 3 Institute of Advanced Research for Sport and Health Science, Ritsumeikan University, Kusatsu, Japan

**Keywords:** Salivary SIgA secretion, Salivary anti-microbial peptide secretion, Acute exercise, Continuous exercise, Interval exercise

## Abstract

**Purpose:**

This study aimed to examine the acute effects of continuous and interval cycling on salivary secretory immunoglobulin A (SIgA) and anti-microbial peptides.

**Methods:**

In a randomized crossover trial, 12 healthy young untrained men (22 ± 1 years) performed two exercise patterns: continuous exercise (CE) for 20-min cycling load equivalent to 70% maximal oxygen uptake (V·O_2max_) and interval exercise (IE) for 20-min cycling exercise, with five sets of 2 min at 50% V·O_2max_ and five sets of 2 min at 90% V·O_2max_. Saliva samples were collected at baseline and post-0 min and post-30 min after exercise. We evaluated salivary SIgA as well as salivary lysozyme, lactoferrin, human β-defensin-2, and LL-37 as the anti-microbial peptides at each time point.

**Results:**

In the CE trial, the salivary SIgA secretion rate decreased immediately after exercise (p = 0.0002). However, in the IE trial, the salivary SIgA secretion rate did not change. In addition, the percentage change in salivary SIgA secretion rate immediately after exercise decreased in the CE as compared to the IE (CE: −37.6 ± 19.7% vs IE: 3.6 ± 33.6%, p = 0.0014). However, salivary lysozyme, lactoferrin, human β-defensin-2, and LL-37 secretion rates did not change from baseline to after exercise in both trials.

**Conclusion:**

These results suggest that salivary SIgA secretion may differ between acute CE and IE, whereas secretory responses to salivary anti-microbial peptides may not differ.

## Introduction

Mucosal epithelial cells in the oral cavity express various immune factors and activate an autonomous innate immune response that prevents the entry of bacteria and viruses through neutralizing and anti-microbial actions. In mucosal secretions, such as those in the oral cavity, immunoglobulin A (IgA) forms a dimer linked by a J chain, such as those in the oral cavity. The J chain-linked dimeric IgA binds to the secretory component to form secretory IgA (SIgA) (Kumar Bharathkar et al. 2020). Salivary SIgA, an antibody secreted by the salivary glands and immune cells, inhibits the proliferation of pathogens through its neutralizing activity (Brandtzaeg 2013). Additionally, anti-microbial peptides such as lysozyme (Lys), lactoferrin (Lac), human β-defensin (HBD)-2, and Cathelicidin (LL-37) have been shown to be secreted by epithelial cells and salivary neutrophils (West et al. 2006). These peptides protect the oral cavity and prevent upper respiratory tract infections by disrupting microbial membranes, inducing bactericidal and antiviral effects, neutralizing pathogenic enzymes (e.g., proteases) and exotoxins released by pathogenic microbes, and regulating the immune system in the oral cavity (Matsuoka et al. 2025). However, an “open window,” defined as a decrease in salivary SIgA levels following acute exercise of higher intensity or longer duration, is associated with upper respiratory tract infections (Nieman et al. 2003; Neville et al. 2008). Therefore, elucidating the immune responses in the oral cavity, including the secretion of salivary SIgA and anti-microbial peptides, following acute exercise is important for understanding the risk of upper respiratory tract infections.

Several studies have reported decreased salivary SIgA secretion after a single session of acute aerobic exercise. In continuous exercise (CE), various exercise intensities and durations have been studied. A previous study reported that the salivary SIgA secretion rate did not change after 30–45 min at 55% maximal oxygen uptake (V·O_2max_) and 60% peak oxygen uptake (V·O_2peak_) intensity (Leicht et al. 2018; Uchino et al. 2024). Conversely, many previous studies have reported 30–60 min at 55–75% V·O_2max_ or > 90 min at 55% V·O_2max_ intensity (Matsubara et al. 2010; Uchino et al. 2024; Murase et al. 2016; Laing et al. 2005), reducing the salivary SIgA secretion rate after exercise. Therefore, the total exercise volume, determined by intensity and duration, reduces salivary SIgA secretion. Recently, interval/intermittent exercise (IE) has been widely used to promote performance and health in athletes and trained or recreationally active individuals (Martin-Smith et al. 2020). Therefore, it is important to elucidate the immune response in the oral cavity to IE. IE comprising 1 min at 100% V·O_2max_ or 90% maximum heart rate (HR_max_), followed by 1–2 min at 30% V·O_2max_ or 30% HR_max_, did not alter the salivary SIgA secretion rate after exercise (Walsh et al. 1999; de Souza et al. 2018). The salivary SIgA secretion rate did not change after sprint exercise for approximately 20 min (Davison 2011). Additionally, a systematic review mentioned to salivary SIgA secretion responses depend on the type of sport, exercise intensity, and exercise duration (Neves et al. 2023). Therefore, the effects of IE on salivary SIgA levels may differ under matched exercise volumes and durations. However, differences in salivary SIgA secretory responses to acute CE and IE remain unclear. Additionally, our recent study demonstrated that 30 min of exercise at 75% V·O_2max_ intensity increased salivary Lac and HBD-2 concentrations, whereas 90 min of exercise at 55% V·O_2max_ intensity increased salivary HBD-2 and LL-37 concentrations (Ito et al. 2024). Thus, as the secretion of salivary anti-microbial peptides after acute exercise varies depending on the intensity and duration of the exercise, the total exercise volume is considered to affect salivary anti-microbial peptide responses. However, the response of salivary anti-microbial peptides to acute IE remains unknown. Therefore, we hypothesized that IE may induce changes that are distinct from the responses to CE because these immune responses in the oral cavity change depending on both intensity and duration.

In this study, we aimed to clarify whether the changes in salivary SIgA and anti-microbial peptide responses differed between CE and IE trials. We compared the secretory responses of salivary SIgA and anti-microbial peptides in healthy young men following acute CE and IE.

## Methods

### Subjects

Twelve healthy young untrained men participants (mean age: 22 ± 1 years; height: 172 ± 6 cm; body weight: 65 ± 4 kg; V·O_2max_: 43 ± 6 ml·kg^−1^·min^−1^) were recruited for this study. The eligibility criteria were as follows: (1) had not performed at least 30 min of physical activity on two or more days per week during the past year, (2) did not have a history of smoking, and (3) did not take regular medications or had experienced chronic illnesses or physical impairments. Before participation, all participants received detailed information on the study’s aims, procedures, and potential risks and provided written informed consent. The study protocol was approved by the Ethics Committee of Ritsumeikan University (approval number: BKC-LSMH-2019-032) and conducted in compliance with the principles outlined in the Declaration of Helsinki.

### Experimental procedures

To determine the exercise intensity for the main test, V·O_2max_ was assessed at least seven days prior. Participants were instructed to avoid high-intensity exercise and alcohol consumption for 24 h before both the V·O_2max_ assessment and the main test. The main test was conducted after a 12 h fasting period during which only water was allowed. The participants arrived at the laboratory in the morning (8:00 AM) and rested for 45 min before beginning the exercise protocol. The exercise loads corresponding to 50, 70 and 90% V·O_2max_ were determined individually for each participant based on the correlation between V·O_2max_ and work rate (Ito et al. 2024; Uchino et al. 2024). Each participant completed two exercise trials separated by at least seven days in a randomized crossover trial. In the CE trial, the participants performed continuous cycling for 20-min at an exercise load equivalent to 70% V·O_2max_, considering the feasibility and physiological comparability with the IE protocol, which included repeated bouts at 90% V·O_2max_. In the IE trial, the participants completed a 20-min cycling exercise consisting of five sets of 2-min at 50% V·O_2max_ followed by five sets of 2-min at 90% V·O_2max_. The total exercise duration was matched between the CE and IE trials, and the exercise volume was approximately equivalent based on oxygen consumption calculations derived from V·O_2max_. The pedaling cadence was standardized at 60 rpm to ensure comparable exercise loads and to allow direct comparison of their physiological effects. This design allowed a practical comparison of the acute effects of continuous and interval cycling under similar overall workload assumptions. Following each exercise session, the participants remained seated and rested for 30 min. Saliva samples were collected at three time points: before exercise (baseline), immediately after exercise (post-0 min), and 30 min after exercise (post-30 min). All trials were conducted in a controlled environment with a stable room temperature of 22.7 ± 0.8 °C and relative humidity of 46.8 ± 13.8% to ensure consistency of the experimental conditions.

### ***Measurement of V·O***_***2max***_

V·O_2max_ was assessed through an incremental cycling exercise test using a cycle ergometer (Model 828E; Monark, Stockholm, Sweden) as previously described (Ito et al. 2024). Before the test, the ergometer’s resistance was adjusted to 60 W, and the participants completed a 5 min warm-up at a cadence of 60 rpm. After warming up, the initial load was set to 60 W, with incremental increases of 15 W every minute until the participants reached their maximal voluntary effort. During the test, heart rate and rating of perceived exertion (RPE) were recorded at each stage. Respiratory gas exchange measurements were performed on a breath-by-breath basis using a gas analyzer (Model AE-310SR D; Minato Medical Science, Osaka, Japan), with values for oxygen uptake (VO_2_), carbon dioxide output (VCO_2_), and respiratory exchange ratio (RER) averaged over 30 s intervals. V·O_2max_ was confirmed if participants achieved at least two of the following criteria: (1) reaching the age-predicted maximum heart rate (220-age ± 5 beats⋅min^−1^), (2) reaching an RER of ≥ 1.1, or (3) reporting an RPE score of ≥ 18. These criteria were applied in accordance with standard guidelines to ensure the reliable determination of V·O_2max_ (American College of Sports Medicine 2000).

### Saliva collection and analysis

Saliva samples were collected by instructing the participants to masticate the paraffin wax (Sakurai Co., Ltd.). Before collection, the participants rinsed their mouths with water for 30 s and then with masticated gum for 60 s to stimulate salivary flow. The saliva flow rate (ml·min^−1^) was determined by dividing the total saliva volume collected over 1 min by the collection time. Salivary concentrations of hemoglobin (BioAssay Systems, Hayward, CA, USA) were used to screen for blood contamination. SIgA (Salimetrics, State College, PA, USA), Lys, Lac (AssayPro, St. Charles, MO, USA), HBD-2 (Phoenix Pharmaceuticals, Burlingame, CA, USA), and LL-37 (Hycult Biotech, Uden, The Netherlands) were quantified using enzyme-linked immunosorbent assays. The absorbance was measured at 450 nm using an xMark microplate spectrophotometer (Bio-Rad Laboratories, Hercules, CA, USA). The secretion rate of each analyte (μg·min^−1^) was calculated by multiplying the salivary concentration (μg·ml^−1^) by the saliva flow rate (ml·min^−1^). The mean coefficients of variation of the assays were as follows: SIgA, 1.6%; Lys, 4.6%; Lac, 2.9%; HBD-2, 0.6%; and LL-37, 2.7%.

### Statistical analysis

Data are presented as mean ± SD. Comparisons of the saliva flow rate, salivary concentrations, and secretion rates of each analyte between the two exercise patterns (CE and IE) were conducted using a two-way repeated-measures ANOVA with two factors: trial (CE and IE) and time point (baseline, post-0 min, and post-30 min). Fisher’s post-hoc test was used to correct for multiple comparisons when significant differences were identified using ANOVA. Statistical significance was set at p < 0.05. The required sample size for this study was calculated using G*Power software (version 3.1.9.6), with an effect size of 0.25 for two-way repeated measures ANOVA (Potvin and Schutz 2000). To detect this effect size with a significance level of 0.05, and a power of 80%, a sample size of 12 participants was deemed sufficient. All statistical analyses were performed using StatView software (version 5.0; SAS Institute, Tokyo, Japan).

## Results

Salivary hemoglobin (as a measure of blood contamination) was not detectable in any saliva samples, hence all participant samples were included in the analysis of SIgA and anti-microbial peptides.

The absolute values of secretion rate in salivary SIgA, Lys, Lac, HBD-2, LL-37 secretion rate, and saliva flow rate did not differ between trials (CE and IE) and showed no significant changes among the three time points (baseline, post-0 min, and post-30 min) (Table 1). Similarly, no differences were observed in the main effects or interactions between trials and time points for the absolute values of salivary SIgA, Lys, Lac, HBD-2, and LL-37 (Table 2).

**Table 1:** Secretion rate of salivary parameters in CE and IE trials

	Trial	Baseline	post-0 min	post-30 min	Interaction p-value
SIgA, μg·min^−1^	CE	263.6 ± 147.9	170.5 ± 100.5	254.2 ± 214.4	0.711
	IE	191.9 ± 97.3	165.9 ± 92.4	226.4 ± 161.6	
Lys, μg·min^−1^	CE	11.3 ± 2.8	8.5 ± 6.5	12.8 ± 3.1	0.868
	IE	8.5 ± 2.4	6.2 ± 4.9	8.2 ± 1.7	
Lac, μg·min^−1^	CE	18.2 ± 8.7	18.4 ± 8.9	24.4 ± 16.7	0.487
	IE	18.5 ± 9.6	12.5 ± 9.9	16.9 ± 8.8	
HBD-2, pg·min^−1^	CE	324.2 ± 65.3	364.6 ± 81.5	381.4 ± 99.3	0.812
	IE	376.3 ± 83.4	319.4 ± 101.7	477.6 ± 204.5	
LL-37, ng·min^−1^	CE	11.5 ± 2.8	10.2 ± 2.5	8.9 ± 4.4	0.860
	IE	10.9 ± 2.5	13.1 ± 7.2	11.8 ± 3.0	
Saliva flow rate, ml·min^−1^	CE	2.9 ± 0.8	2.0 ± 0.8	2.7 ± 0.8	0.935
	IE	2.5 ± 0.9	1.8 ± 0.9	2.5 ± 1.0	

Data are presented as mean ± SD. SIgA: Secretory immunoglobulin A, Lys: Lysozyme, Lac: Lactoferrin, HBD-2: Human β-defensin-2, LL-37: Cathelicidin

CE: Continuous exercise, IE: Interval exercise

**Table 2:** Concentrations of Salivary parameter in CE and IE trials

	Trial	Baseline	post-0 min	post-30 min	Interaction p value
SIgA, μg·ml^−1^	CE	100.2 ± 63.7	109.2 ± 84.0	99.2 ± 75.4	0.751
	IE	90.2 ± 57.2	132.2 ± 101.0	101.0 ± 71.2	
Lys, μg·ml^−1^	CE	3.7 ± 2.6	4.5 ± 2.8	4.4 ± 3.2	0.959
	IE	3.0 ± 2.3	3.8 ± 2.7	3.3 ± 2.2	
Lac, μg·ml^−1^	CE	6.6 ± 2.6	11.6 ± 7.9	9.2 ± 6.0	0.223
	IE	7.6 ± 2.8	7.8 ± 2.7	7.1 ± 3.8	
HBD-2, pg·ml^−1^	CE	114.2 ± 72.1	178.8 ± 100.0	125.9 ± 90.2	0.548
	IE	144.0 ± 94.0	153.3 ± 101.5	162.0 ± 49.2	
LL-37, ng·ml^−1^	CE	3.9 ± 2.8	4.8 ± 3.1	3.3 ± 1.7	0.934
	IE	4.2 ± 3.0	5.4 ± 1.8	4.4 ± 2.8	

Data are presented as mean ± SD. SIgA: Secretory immunoglobulin A, Lys: Lysozyme, Lac: Lactoferrin, HBD-2: Human β-defensin-2, LL-37: Cathelicidin

CE: Continuous exercise, IE: Interval exercise

**Fig. 1:**
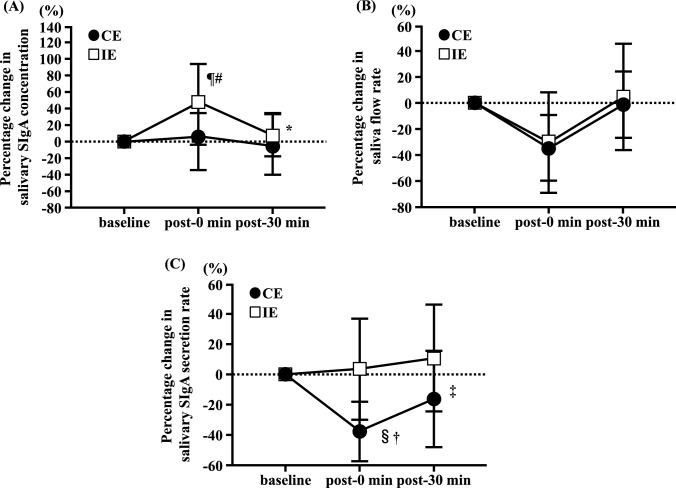
Effects of continuous and interval cycling on the percentage change in salivary SIgA concentration (**A**), saliva flow rate (**B**), and salivary SIgA secretion rate (**C**) from baseline in healthy untrained young men. (n = 12). Data are presented as mean ± SD. SIgA: Secretory immunoglobulin A. ¶p < 0.05 vs. CE at post-0 min. ^#^p < 0.01 vs. baseline in IE. *p < 0.01 vs. post-0 min in IE. ^§^p < 0.01 vs. IE at post-0 min. ^†^p < 0.01 vs. baseline in CE. ^‡^p < 0.01 vs. post-0 min in CE

The percentage change in the salivary SIgA concentration showed an interaction effect (interaction, p = 0.034; Fig. 1A). In the CE trial, no differences were observed in the percentage change in salivary SIgA concentrations from baseline to post-30 min (Fig. 1A). In the IE trial, the percentage change in salivary SIgA concentration at post-0 min was significantly higher than that at baseline (p < 0.01; Fig. 1A), and significantly lower at post-30 min than at post-0 min (p < 0.05; Fig. 1A). Furthermore, the percentage change in salivary SIgA concentration was significantly lower in the CE trial than in the IE trial at post-0 min (p < 0.01; Fig. 1A). Although no significant differences in the percentage change in the saliva flow rate were observed between the trials, it was significantly lower post-0 min than at baseline (main effect of time, p < 0.0001; Fig. 1B). There was a significant interaction effect on salivary SIgA secretion rate (interaction: p = 0.020; Fig. 1C). In the IE trial, no significant differences were observed in the percentage change in salivary SIgA secretion rates from baseline to post-30 min (Fig. 1C). In the CE trial, the percentage change in the salivary SIgA secretion rate at post-0 min was significantly lower than that at baseline (p < 0.01; Fig. 1C) and significantly higher at post-30 min than at post-0 min (p < 0.05; Fig. 1C). Additionally, the percentage change in the salivary SIgA secretion rate at post-0 min was significantly lower in the CE trial than in the IE trial (p < 0.01; Fig. 1C).

**Fig. 2:**
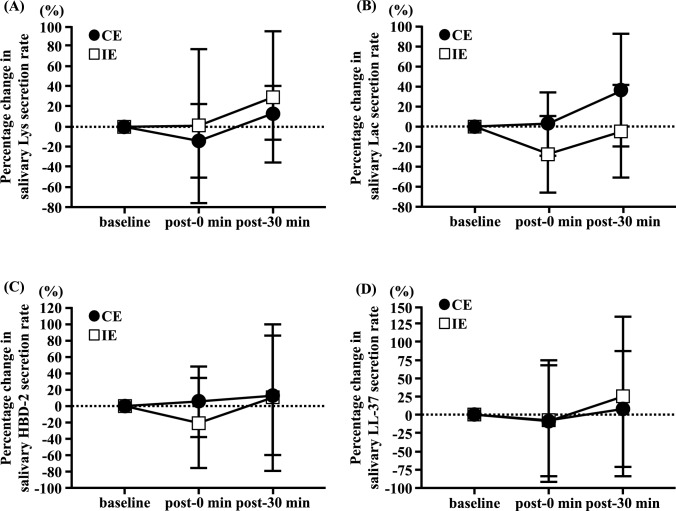
Effects of continuous and interval cycling on the percentage change in salivary Lys secretion rate (**A**), salivary Lac secretion rate (**B**), salivary HBD-2 secretion rate (**C**), and salivary LL-37 secretion rate (**D**) from baseline in healthy untrained young men (n = 12). Data are presented as mean ± SD. Lys: Lysozyme, Lac: Lactoferrin, HBD-2: Human β-defensin-2, LL-37: Cathelicidin

The percentage changes in salivary Lys, Lac, HBD-2, and LL-37 secretion rates did not significantly change from baseline to post-30 min in the CE and IE trials (Fig. 2A–D). The percentage changes in salivary Lac and HBD-2 concentrations showed an interaction effect (interaction: Lac, p = 0.049; HBD-2, p = 0.027; Table 3). In the IE trial, no significant differences were observed in the percentage changes in salivary Lac and HBD-2 concentrations from baseline to post-30 min (Table 3). In the CE trial, the percentage changes in salivary Lac and HBD-2 concentrations were significantly higher post-0 min than those at baseline (P < 0.01; Table 3), and the percentage change in salivary HBD-2 concentration at post-30 min was significantly lower than at post-0 min (p < 0.05; Table 3). Furthermore, the percentage changes in the salivary Lac and HBD-2 concentrations in the CE trial were significantly higher than those in the IE trial at post-0 min (Lac, p < 0.05; HBD-2, p < 0.01; Table 3). However, no significant interaction effect was observed for the percentage change in salivary Lys and LL-37 concentrations from baseline to post-30 min among the trials (Table 3).

**Table 3:** Percentage change in concentration of salivary anti-microbial peptides in CE and IE trials

	Trial	Post-0 min	Post-30 min	Interaction p value
Lys, %	CE	31.3 ± 30.4	19.8 ± 30.6	0.960
	IE	35.8 ± 43.1	23.7 ± 39.3	
Lac, %	CE	68.8 ± 58.1^†#^	35.5 ± 45.8	0.049
	IE	10.8 ± 45.7	− 1.5 ± 47.5	
HBD-2, %	CE	65.7 ± 51.5^‡#^	9.7 ± 54.0*	0.027
	IE	8.0 ± 39.3	− 0.5 ± 42.6	
LL-37, %	CE	32.5 ± 63.1	4.0 ± 63.6	0.731
	IE	18.9 ± 44.9	11.7 ± 57.3	

Data are presented as mean ± SD. Lys: Lysozyme, Lac: Lactoferrin, HBD-2: Human β-defensin-2, LL-37: Cathelicidin

CE: Continuous exercise, IE: Interval exercise

^†^p < 0.05, ^‡^p < 0.01 vs. IE at post-0 min; ^#^p < 0.01 vs. baseline in CE; *p < 0.05 vs. post-0 min in CE

## Discussion

In this study, the absolute values of secretion rates and concentrations of salivary SIgA, Lys, Lac, HBD-2, LL-37, and saliva flow rate did not differ between the CE and IE trials. The percentage change from baseline to after exercise was examined to adjust for the effects of individual differences. Our results demonstrate that in the IE trial comprising 20-min of five sets of 2-min at 50% V·O_2max_ and five sets of 2-min at 90% V·O_2max_, the percentage change in salivary SIgA, Lys, Lac, HBD-2, and LL-37 secretion rate did not exhibit a decrease from baseline to immediately post-exercise and 30-min post-exercise. Conversely, in the CE trial for 20-min at 70% V·O_2max_ exercise intensity, the percentage change in salivary SIgA secretion rate decreased immediately post-exercise compared to that at baseline and was lower than that observed in the IE trial immediately post-exercise. However, the percentage changes in salivary Lys, Lac, HBD-2, and LL-37 secretion rates did not change from baseline to immediately after exercise. These findings suggest that salivary SIgA secretion may differ between acute CE and IE, whereas the secretory responses of salivary anti-microbial peptides may not differ.

A previous study reported that CE with higher intensity or longer duration induced an “open window,” a transient immunosuppressed state after exercise (Nieman et al. 2003). Although a few studies have shown that 30–45 min of exercise at 55% V·O_2max_ and 60% V·O_2peak_ intensity did not alter the salivary SIgA secretion rate (Leicht et al. 2018; Uchino et al. 2024), several studies have reported that the salivary SIgA secretion rate decreased after 30–60 min at 55%–75% V·O_2max_ intensity or > 90 min at 55% V·O_2max_ intensity (Matsubara et al. 2010; Uchino et al. 2024; Murase et al. 2016; Laing et al. 2005). In this study, the salivary SIgA secretion rate decreased after 20 min of CE, indicating that transient immunosuppression occurred, as reported in previous studies. However, salivary SIgA secretion did not decrease after IE treatment. During CE in this study, the mean heart rate was 161 ± 18 beats⋅min^−1^, whereas during IE, the mean heart rate was 172 ± 15 beats⋅min^−1^ in the 2-min periods at 90% V·O_2max_ and was 147 ± 18 beats⋅min^−1^ in the 2-min periods at 50% V·O_2max_. Therefore, the exercise intensity during the 2-min period at 90% V·O_2max_ during IE was higher than that during CE. The 2-min period at 90% V·O_2max_ in IE was too short to inhibit salivary SIgA secretion, and the exercise load shifted before inhibition could occur. Consequently, suppression of salivary SIgA secretion in IE may not occur to the same extent as in CE. Previous studies have shown that IE for 10–20 min, consisting of 1 min at 100% V·O_2max_ or 90% HR_max_ and 1–2 min at 30% V·O_2max_ or 30% HR_max_, did not alter the salivary SIgA secretion rate after exercise (Walsh et al. 1999; de Souza et al. 2018). Therefore, acute CE induces transient immunosuppression, whereas acute IE may preserve the mucosal immune responses by maintaining a salivary SIgA response.

In this study, differences in exercise patterns did not affect the saliva flow rate. Moreover, salivary anti-microbial peptide concentrations are associated with the onset of upper respiratory tract infections; therefore, it is important to evaluate these concentrations during acute exercise (Usui et al. 2012). In the present study, salivary Lys and LL-37 concentrations did not change regardless of the exercise pattern, but salivary Lac and HBD-2 concentrations increased after CE compared with that after IE. In our recent study, salivary Lac and HBD-2 concentrations increased after 30 min of exercise at 75% V·O_2max_, whereas salivary Lys and LL-37 concentrations did not change (Ito et al. 2024). Therefore, among the salivary anti-microbial peptides, salivary Lac and HBD-2 concentrations increased after CE. However, in IE, salivary Lys and LL-37 concentrations did not change. In this study, the salivary SIgA secretion rate decreased after CE. A previous study reported that the increase in salivary anti-microbial peptide concentrations after exercise is a response to changes in other salivary immune factors, such as salivary SIgA (Davison et al. 2009). Therefore, increased concentrations of salivary anti-microbial peptides may compensate for the transient decrease in salivary SIgA secretion.

Salivary SIgA is produced by immune cells and secreted by the salivary glands (Brandtzaeg 2013). Additionally, salivary anti-microbial peptides are produced by both epithelial cells and salivary neutrophils, and their secretion is induced by local inflammatory stimuli and pathogen recognition (West et al. 2006). The autonomic nervous system regulates protein secretion in the saliva, and salivary gland cells are affected by the sympathetic and parasympathetic nervous systems (Carpenter et al. 2004). Previous studies have shown that the activation of sympathetic nervous activity (Kindermann et al. 1982) and the promotion of inflammatory cytokine (IL-6 and TNF-α) secretion (Ploeger et al. 2012) are higher in CE than in IE. Furthermore, the activation of the sympathetic nervous system suppresses immune cell activity, leading to an increase in inflammatory cytokines (Yamakawa et al. 2009; Campos-Rodríguez et al. 2013). Therefore, the differences in salivary SIgA and anti-microbial peptide secretory responses to CE observed in this study may be related to differences in production sources and secretory mechanisms.

Habitual IE is widely used as an effective exercise to improve athletic performance and promote health (Martin-Smith et al. 2020). In a previous study, athletes performed 20–30 min of IE at > 90% HR_max_ over six weeks, and V·O_2max_ increased after interval training (Franch et al. 1998). Additionally, in trained/recreationally active men, interval training at 90–95% HR_max_ improved V·O_2max_ more than endurance training or continuous exercise training (Helgerud et al. 2007). Traditionally, a single bout of acute CE has been shown to enhance immune function, whereas acute IE induces transient immunosuppression (Gleeson 2007; Khammassi et al. 2020). However, in this study, which compared the effects of CE and IE on oral immune responses, IE did not reduce the immune responses compared to that with CE. Therefore, 20-min of IE may represent a useful exercise that does not impair immune function.

This study had several limitations. First, this study did not allow fluid intake during exercise or the measurement of changes in body weight before and after exercise. Previous studies have reported that when body weight decreases by more than 3% before and after exercise, dehydration can contribute to a reduction in the saliva flow rate (Walsh et al. 2004). In this study, a decrease in saliva flow rate observed after exercise and a decrease in total body water may have affected this phenomenon. Future studies should adopt an experimental protocol designed to minimize the reduction in the saliva flow rate. Second, the method of collecting saliva using an unstimulated passive drool in the previous study differed from the stimulated sampling using gum in this study, and this difference may have affected the parameters measured during exercise. Therefore, it is necessary to consider non-stimulatory methods in the future. Third, because salivary parameters were adjusted by salivary osmolality, secretion rate, and absolute concentration in previous studies, further investigation of salivary parameters during acute exercise, considering adjustment by salivary osmolality, is necessary.

In conclusion, this study revealed that salivary SIgA secretion decreased after exercise in CE trial, whereas salivary SIgA, Lys, Lac, HBD-2, and LL-37 secretion did not change after IE. Thus, in healthy, young, untrained men, salivary SIgA secretion may differ between acute CE and IE. Some anti-microbial peptides remain stable, while other anti-microbial peptides respond in a slightly different way.

## Data Availability

The datasets generated in this study are available from the corresponding author upon request.

## Abbreviations

CE: Continuous exercise
HBD-2: Human β-defensin-2
IE: Interval/intermittent exercise
Lac: Lactoferrin
LL-37: Cathelicidin
Lys: Lysozyme
SIgA: Secretory Immunoglobulin A
V·O_2max_: Maximal oxygen uptake
